# Prevalence and Predictors of Radiographically Apparent Upper Urinary Tract Urolithiasis in Eight Dog Breeds Predisposed to Calcium Oxalate Urolithiasis and Mixed Breed Dogs

**DOI:** 10.3390/vetsci9060283

**Published:** 2022-06-09

**Authors:** Alexis M. Hoelmer, Jody P. Lulich, Aaron K. Rendahl, Eva Furrow

**Affiliations:** 1 Department of Veterinary Clinical Sciences, College of Veterinary Medicine, University of Minnesota, St. Paul, MN 55108, USA; hoelm009@umn.edu (A.M.H.); lulic001@umn.edu (J.P.L.); rend0020@umn.edu (A.K.R.); 2 C339 Veterinary Medical Center, 1352 Boyd Ave, St. Paul, MN 55108, USA

**Keywords:** urinary stones, nephroliths, ureteroliths, kidney stones

## Abstract

Data on upper urinary tract (UUT) uroliths in dogs are important to understanding their etiology. The aim of this retrospective case-control study was to determine the prevalence and identify predictors of radiographically apparent UUT uroliths in dog breeds at increased risk for calcium oxalate uroliths (CaOx risk breeds) and mixed breed dogs. Radiologist reports of three-view abdominal radiographs were reviewed from 251 purebred dogs of 8 CaOx risk breeds and 68 mixed breed dogs. UUT uroliths were more common in CaOx risk breeds than mixed breed dogs (23% versus 6%, respectively; OR = 4.8, 95% confidence interval [CI] 1.7–18.9, *p* < 0.001). UUT uroliths were more common in dogs with lower urinary tract (LUT) uroliths (predominantly calcium-containing) than those without (41% versus 5%, respectively; OR = 13.6, 95% CI 6.3–33.1, *p* < 0.001), and LUT uroliths predicted the presence of UUT uroliths in the multivariable regression (OR = 6.5, 95% CI 2.8–16.7, *p* < 0.001). Increasing age (*p* < 0.001) and lower body weight (*p* = 0.0016) were also predictors of UUT urolith presence in the multivariable regression. The high prevalence of UUT uroliths in dogs with LUT uroliths supports a shared mechanism for their formation.

## 1. Introduction

Data from urolith analysis centers provide insight into patient risk factors for uroliths, which are important to their clinical recognition and for an understanding of pathophysiology [1,2,3,4]. However, while urolith centers are a robust data source, the data are biased by the submissions received. This limitation is of particular importance for research on upper urinary tract (UUT) uroliths in dogs. Greater than 75% of UUT uroliths in dogs are non-obstructive [5], and removal is not recommended in the absence of pain or other complications [6]. As a result, UUT uroliths are infrequently submitted to urolith analysis centers, comprising only 1–2% of all canine urolith submissions [1,4]. Data on UUT uroliths are needed from other sources that do not rely on urolith removal for diagnosis.

Further, different pathologic mechanisms are theorized to contribute to the formation of uroliths within the UUT versus the lower urinary tract (LUT). For example, in humans, UUT uroliths often adhere to renal interstitial mineralization, which is thought to serve as a nidus [7]. In contrast, the combination of urine supersaturation and urine retention is important to the formation of LUT uroliths [8,9]. Whether UUT and LUT urolith forming dogs represent distinct populations with diverse etiologies is unknown.

The objective of this study was to determine the prevalence and predictors of radiographically apparent UUT uroliths in breeds predisposed to calcium oxalate (CaOx) urolithiasis and mixed breed dogs. We chose to focus on breeds at increased risk for CaOx urolithiasis because this is one of the most common compositions for both UUT and LUT uroliths in dogs [1,4]. Predictors of UUT uroliths evaluated were active or historic LUT urolithiasis, sex, age, breed, and body weight. We hypothesized that UUT uroliths are common in breeds at increased risk for CaOx urolithiasis and associated with LUT urolithiasis, male sex, older age, and lower body weight.

## 2. Methods

### 2.1. Study Population

This was a retrospective study at the University of Minnesota Veterinary Medical Center. The electronic medical record database was searched for dogs examined between 1 May 2016 and 30 April 2020 that had a charge code for three-view abdominal radiographs. Purebred dogs were selected of the top five breeds predisposed to CaOx uroliths in two urolith analysis centers in the United States [2,3], including the Bichon Frise, Lhasa Apso, Miniature Schnauzer, Pomeranian, and Shih Tzu. Since these are all toy or small breeds (standard weight < 10 kg), three additional at-risk breeds were selected to represent medium-to-large breeds, including the Doberman Pinscher, Standard Poodle, and Standard Schnauzer [10,11]. We refer to these eight breeds as the “CaOx risk breeds.” Mixed breed dogs were included as a reference group; these were dogs where the breed was coded as “mixed breed dog” in the electronic medical record rather than as a cross or mix of a specific breed.

### 2.2. Medical Record Data Extracted

Breed, sex, age, and weight were recorded. The indication for the radiographs was recorded and categorized based on whether it included known or suspected urolithiasis or if the indication was unrelated to urolithiasis. If multiple sets of abdominal radiographs were available for a dog, the most recent was selected for review. The radiologist reports were reviewed and categorized into two groups based on the presence or absence of UUT mineralization. Language varied among reports, some using the term “nephroureterolith” and others stating “mineral focus” or “mineralization” with UUT urolithiasis as the top differential. The radiologist report and the medical history were also reviewed to determine if the dog had active or historic LUT uroliths. If available, urolith analysis was recorded, and the composition was characterized as pure CaOx (100%), part calcium (mixed or compound with a CaOx or calcium phosphate component), or other. Urolith compositions were determined by analysis at the Minnesota Urolith Center (polarized light microscopy and infrared spectroscopy).

### 2.3. Statistical Analysis

Data distribution and normality of continuous variables (age, weight) were evaluated by inspection of Q-Q plots and the Shapiro-Wilk test. These variables did not follow the normal distribution and are reported as median (range). The Wilcoxon rank-sum test was used to compare age and weight between CaOx risk and mixed breed dogs and between dogs with and without UUT urolithiasis. Chi-square tests were used to compare sex between CaOx risk and mixed breed dogs and to compare the proportion of dogs with UUT urolithiasis between dogs with and without LUT urolithiasis (active or historic), males and females, and CaOx risk versus mixed breed dogs. Odds ratios and 95% confidence intervals were calculated for the proportions. Multivariable logistic regression was performed to test sex (male versus female), age (years), breed, LUT urolith status (active or historic urolithiasis versus none), and weight (kg) as predictors of UUT urolithiasis. The significance of each model predictor was determined using Type II tests (ANCOVA). Odds ratios and 95% confidence intervals were calculated for the increment value of each predictor. All analyses were performed using R software for statistical computing (R, version 4.1.0. R Core Team (2021). R Foundation for Statistical Computing, Vienna, Austria. http://www.r-project.org, accessed 18 May 2021) using packages car, MASS, oddsratio and, for graphing, ggplot2 [12,13,14,15]. A *p* value < 0.05 was used to define statistical significance.

## 3. Results

### 3.1. General Study Population Characteristics

The database search identified 319 dogs that met inclusion criteria. The study group included 68 mixed breed dogs, 65 Miniature Schnauzers, 58 Shih Tzus, 38 Bichon Frise, 28 Standard Poodles, 20 Dobermans, 20 Pomeranians, 16 Lhasa Apsos, and 6 Standard Schnauzers. There were 188 male (168 neutered, 19 intact, and 1 unreported neuter status) and 131 female (124 spayed and 7 intact) dogs. The median age of the study dogs was 8.8 years (0.2–15.8 years), and the median weight was 8.9 kg (1.6–52.8 kg). The mixed breed dogs were younger, weighed more, and had a lower proportion of LUT urolithiasis than the CaOx risk breed dogs, but the sex distribution did not differ (Table 1).

The indication for abdominal radiographs was for known or suspected urolithiasis in 131 dogs (11 mixed breed and 120 CaOx risk breed). An additional 18 CaOx risk breed dogs were being screened as potential controls (no urolithiasis history) for genetic and metabolic studies on CaOx urolithiasis in dogs. One hundred and seventy dogs had radiographs taken for a reason unrelated to urolithiasis, including 145 for suspected gastrointestinal disease and 25 for other causes (<5 dogs each).

### 3.2. Prevalence and Composition of LUT Uroliths

One hundred and thirty of the 319 dogs (41%) had active or historic LUT urolithiasis, including 58% (115/197) of small CaOx risk breed dogs, 9% (5/54) of medium-to-large CaOx risk breed dogs, and 15% (10/68) of mixed breed dogs. One hundred and twenty-two of the 130 dogs (94%) with active or historic LUT urolithiasis had known or suspected urolithiasis as the indication for abdominal radiographs. Urolith composition was available for 58% (75/130). Types consisted of 68% (51/75) pure CaOx, 27% (20/75) part calcium, and 5% (4/75) other mineral types (2 struvite, 1 urate, and 1 mixed silica and urate).

### 3.3. Prevalence of UUT Uroliths and Association with Patient Factors

Sixty-two of the 319 dogs (19%) had radiographic evidence of UUT urolithiasis, including 57 dogs with nephroliths only, 3 with nephroliths and ureteroliths, and 2 with ureteroliths only. Fifty-three of the 62 dogs (85%) with UUT urolithiasis had known or suspected urolithiasis as the indication for abdominal radiographs. Only one UUT urolith was removed and analyzed; it was a ureterolith composed of 95% CaOx and 5% calcium phosphate.

**Figure 1:**
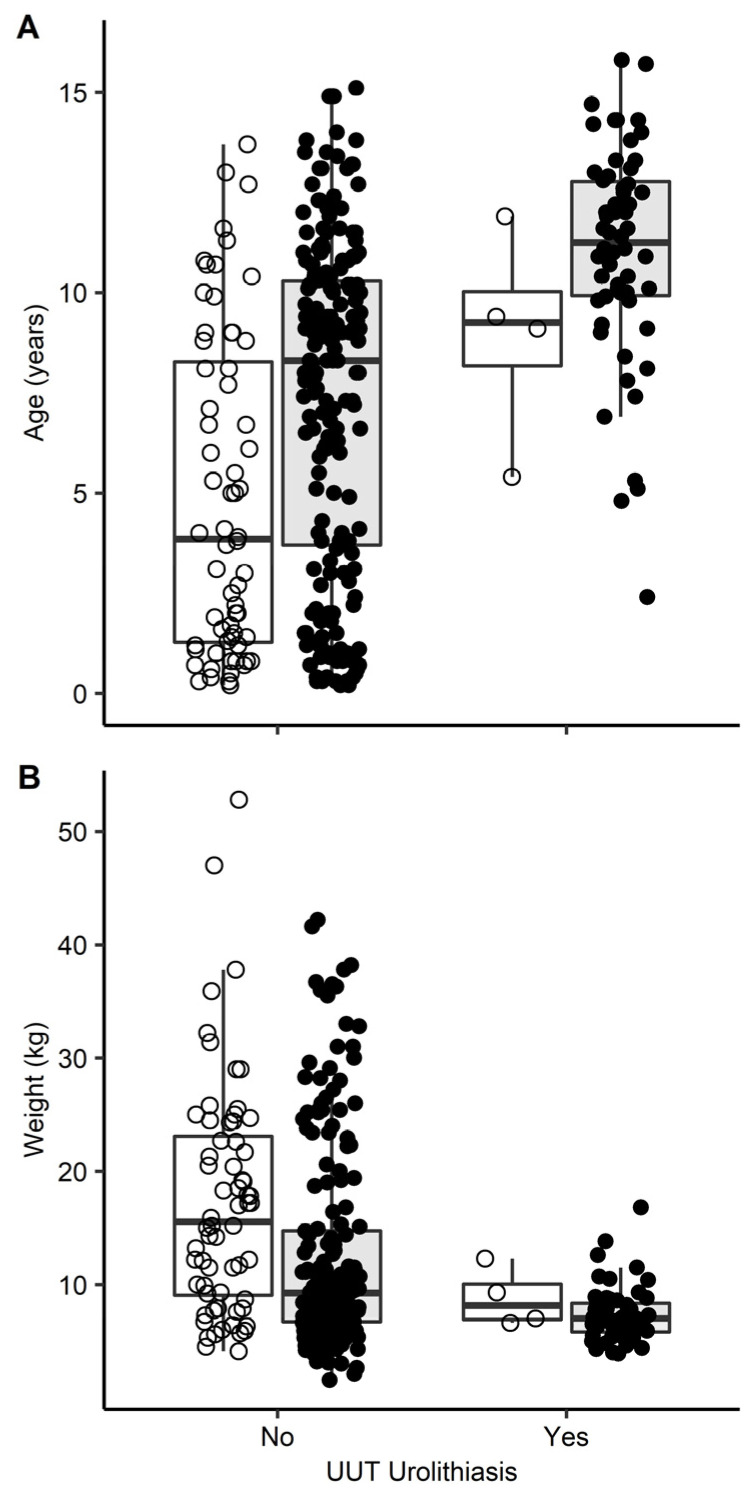
Box and whisker plots of (**A**) age and (**B**) weight for 319 dogs with abdominal radiographs reviewed for upper urinary tract (UUT) urolithiasis. Dogs of breeds at increased risk for calcium oxalate urolithiasis (*n* = 251) are represented with closed dots and grey shaded boxes and mixed breed dogs (*n* = 68) are represented with open dots and open boxes. The boxes represent the interquartile range, and the whiskers represent 1.5 times the interquartile range. Dogs with UUT uroliths were older and weighed less than those without (*p* < 0.001 for both comparisons).

UUT uroliths were more common in the CaOx risk breeds (23%, 58/251) compared with mixed breed dogs (6%, 4/68; Table 2). None of the 54 CaOx risk medium-to-large breed dogs had radiographic evidence of UUT urolithiasis. The prevalence of UUT uroliths did not differ by sex (Table 2). There were significant differences in age and weight between dogs with and without UUT urolithiasis (Figure 1). Dogs with UUT urolithiasis were older (median 11.1 years, range 2.4–15.8 years) than dogs without UUT urolithiasis (median 7.7 years, range 0.2–15.1; *p* < 0.001). Dogs with UUT urolithiasis were also smaller (median 7.0 kg, range 3.9–16.8 kg) than dogs without urolithiasis (median 10.1 kg, range 1.6–52.8 kg; *p* < 0.001).

Forty-one percent (53/130) of dogs with active or historic LUT urolithiasis had UUT urolithiasis compared with 5% (9/189) of dogs without LUT urolithiasis (Table 2). In a subanalysis of only the small CaOx risk breeds, UUT uroliths were present in 81% (47/58) of those with active or historic LUT uroliths compared to 11% (7/62) without (OR = 7.1, 95% CI 2.9–20.1, *p* < 0.001). Overall, 85% (53/62) of UUT uroliths across all study breeds were in dogs with active or historic LUT urolithiasis.

Predictors of UUT uroliths were analyzed further with multivariable regression (Table 3). Increasing age, decreasing weight, and active or historic LUT urolithiasis were predictors of UUT urolithiasis. The breed was not identified as a predictor of UUT urolith risk in the regression analysis.

## 4. Discussion

In this study of eight CaOx risk breed and mixed breed dogs, radiographically apparent UUT uroliths were common, detected in 19% of dogs overall. Active or historic LUT urolithiasis was the strongest predictor of UUT urolithiasis, followed by older age and smaller body weight. Neither sex nor individual breed was found to affect the odds of UUT urolithiasis in a multivariable model that accounted for LUT urolithiasis, age, and weight. None of the three medium-to-large CaOx risk breeds had UUT urolithiasis identified.

The relatively high prevalence of UUT uroliths in this study suggests that submissions to urolith centers grossly underestimate their frequency relative to LUT uroliths. Only 1 of 62 dogs (1.6%) with UUT urolithiasis in this study had the urolith removed and analyzed for composition, whereas 58% of the LUT urolithiasis underwent compositional analysis. Similarly, a previous study found UUT uroliths in 8 of 24 Miniature Schnauzers undergoing abdominal radiography and ultrasonography; none were removed and analyzed [16]. It is important to note that 41% of the dogs in the present study had active or historic LUT urolithiasis, and nearly the same proportion had urolithiasis as the indication for abdominal radiographs. The study group was also relatively old, with a median age of around 9 years. These factors likely created a strong bias towards detecting a higher prevalence of UUT uroliths than what might be found in a general population of dogs. For comparison, the prevalence of a urolithiasis diagnosis in dogs presenting to primary care hospitals in the United States is only 0.03% [17].

The increased risk for UUT urolithiasis in dogs with LUT urolithiasis supports shared pathophysiology for urolith formation in these anatomic locations. It is also possible that some uroliths form first in the UUT and pass into the LUT, where they grow to a size that is more easily detected. UUT uroliths were present in 41% of all study dogs with LUT urolithiasis and 81% of the small CaOx risk breeds with LUT urolithiasis. This again parallels findings in the aforementioned study of Miniature Schnauzers undergoing abdominal radiography and ultrasonography. In that study, six out of eight of the dogs with UUT uroliths had active or historic LUT uroliths [16]. Cats likewise have a high co-occurrence of UUT and LUT uroliths, with one-third of cats with UUT uroliths having concurrent LUT uroliths [18]. In humans, LUT uroliths are relatively uncommon compared to UUT uroliths, representing less than 10% of urolithiasis cases [19]. However, the human UUT and LUT urolithiasis populations are interconnected, with both diagnoses running in the same families [19]. There is also familial overlap between predispositions for UUT and LUT urolithiasis in dogs; the Lhasa Apso, Bichon Frise, Miniature Schnauzer, Shih Tzu, and Yorkshire Terrier are at increased risk for both diagnoses [2,3,20,21].

Of the uroliths analyzed, 95% contained calcium, either as pure CaOx (68%) or mixed or compound with a calcium component. This characteristic of the study group should be considered when translating the findings to other populations. The frequency of UUT and LUT urolith co-occurrence likely differs by urolith composition, and results might not translate well to non-calcium-containing urolith types. For example, a greater proportion of xanthine urolith submissions (16%) from dogs come from the UUT than proportions for all other urolith types (which range from 1–3%) [4].

The median age of dogs with UUT urolithiasis in this study was 11 years, and increasing age was a risk factor for UUT uroliths. This is greater than the mean age of 7–8 years for all canine UUT urolith submissions to an analysis center [20]. Differences might again be due to the urolith composition; while the mean age of dogs with CaOx UUT uroliths is 9 years, the mean age of dogs with purine UUT uroliths is lower at 5 years [21]. Age is a major risk factor for CaOx urolithiasis in dogs, with a median of 8–9 years at the time of urolith analysis [3,10]. A limitation of the present study is that its retrospective nature impedes the ability to detect the age when UUT uroliths first developed. Some dogs had multiple sets of abdominal radiographs, in which case we used the most recent encounter for consistency. Thus, the age reported for dogs with UUT urolithiasis in this study is likely biased towards an older age.

Dogs with greater body weight were found to be at a lower risk for UUT urolithiasis. The median weight of dogs without UUT uroliths was 10.1 kg compared to 7.7 kg for those with UUT urolithiasis, and no UUT uroliths were detected in dogs ≥ 17 kg. Greater body weight is also associated with a lower risk for composition-confirmed CaOx urolithiasis in dogs, where the mean weight of controls is 13.9 kg compared to 8.2 kg for cases [17]. Body weight differs by breed and might reflect differences in ancestry that might impact urolithiasis risk rather than a direct effect of weight. However, we identified body weight as a predictor of UUT uroliths in the multivariable models that accounted for the breed, sex, and other variables. Body weight is impacted by both height and body condition. In men and women, height has an inverse relationship with UUT uroliths, while body mass index has the opposite effect and increases risk [22]. An overweight body condition is also associated with CaOx urolithiasis in dogs [10,23]. Height and body condition scores were not available in the present study and, such as in humans, might have opposing effects on UUT urolithiasis risk in dogs that warrant investigation. The reason for the protective effect of higher body weight against UUT urolithiasis in dogs might be anatomical. If body weight correlates with ureteral diameter or length, it might affect the likelihood of spontaneous passage of smaller UUT uroliths. Ureteral diameter in dogs varies from 1–4 mm [24,25,26], though reports on its correlation to body weight are lacking. Another possibility is that UUT uroliths might be more difficult to radiographically identify in larger dogs, creating an appearance of a lower prevalence. Other imaging techniques, such as computed tomography, could help determine if the reduced risk for UUT uroliths with increasing body weight is true or an artifact of the lower sensitivity of radiography in larger dogs.

Male sex was not identified as a predictor of UUT urolithiasis in the study dogs. Sex distribution is affected by urolith composition. Female dogs are overrepresented for UUT uroliths composed of struvite, but males and females comprise similar proportions of UUT uroliths composed of CaOx [21]. The sex distribution for CaOx urolithiasis overall is different, with a strong male predisposition [2,3]. These findings are consistent with the theory that the anatomy of the penile urethra (tapering of the penile urethra and presence of an os penis) results in a lower rate of spontaneous urolith passage in male dogs compared with females, rather than a sex-dependent effect on CaOx urolith formation, which would be expected to equally affect UUT and LUT uroliths.

A limitation of this study is that radiography is not the most sensitive imaging modality for UUT uroliths [27]. Thus, our results reflect the prevalence of radiographically apparent UUT uroliths rather than the absolute prevalence in the study dogs. Another limitation of radiography is that mineralization in the area of the kidney is not definitive for UUT urolithiasis. We cannot determine whether the mineralization in some cases was due to nephrocalcinosis, which might have unique pathogenic mechanisms [28]. Finally, radiography does not distinguish obstructing from non-obstructing UUT uroliths.

In conclusion, radiographically apparent UUT uroliths are common in small breed, mature adult dogs with active or historic LUT urolithiasis. This suggests that dogs with urolithiasis anywhere in the urinary tract should be considered at risk for the development of uroliths in additional anatomic sites. The results of this study help prepare veterinarians that they will often detect UUT uroliths when performing abdominal radiography in similar populations of dogs. The results should also inform research on urolith pathogenesis.

## Figures and Tables

**Table 1:** Sex distribution, age (median) and weight (median) for the study dogs, including 68 mixed breed dogs and 251 dogs of breeds reported having increased risk for calcium oxalate (CaOx) uroliths.

Variable	Mixed Breed	CaOx Risk Breed	*p* Value
Male sex (proportion)	0.60 (41/68)	0.59 (147/251)	0.91
LUT urolithiasis (proportion)	0.15 (10/68)	0.48 (120/131)	<0.001
Age (yr)	4.1 (0.1–13.7)	9.1 (0.2–15.8)	<0.001
Weight (kg)	15.1 (4.1–52.8)	8.3 (1.6–42.2)	<0.001

LUT, lower urinary tract.

**Table 2:** Prevalence of radiographically apparent upper urinary tract (UUT) uroliths and their association with patient variables in 319 dogs, including 68 mixed breed dogs and 251 dogs of breeds reported to have increased risk for calcium oxalate (CaOx) uroliths.

Variable	UUT Uroliths Present, Proportion (#/Total)	OR	95% CI	*p* Value
Female sex (referent)	0.21 (27/131)	--	--	--
Male sex	0.19 (35/188)	0.88	0.49–1.6	0.77
Mixed breed (referent)	0.06 (4/68)	--	--	--
CaOx risk breed	0.23 (58/251)	4.8	1.7–18.9	<0.001
Lhasa Apso	0.44 (7/16)			
Bichon	0.39 (15/38)			
Shih Tzu	0.29 (17/58)			
Pomeranian	0.25 (5/20)			
Miniature Schnauzer	0.22 (14/65)			
Doberman	0.0 (0/20)			
Standard Schnauzer	0.0 (0/6)			
Standard Poodle	0.0 (0/28)			
No LUT urolithiasis	0.05 (9/189)	--	--	--
LUT urolithiasis	0.41 (53/130)	13.6	6.3–33.1	<0.001
Total	0.19 (62/319)			

LUT, lower urinary tract; --, not applicable for the referent.

**Table 3:** Multivariable regression analysis of predictors of radiographically apparent upper urinary tract urolithiasis in 319 dogs, including 68 mixed breed dogs and 251 dogs of breeds reported to have increased risk for calcium oxalate (CaOx) uroliths.

Predictor	Coefficient Estimate	Standard Error	Odds Ratio (95% CI)	*p* Value
Male sex	−0.15	0.38	0.9 (0.4–1.8)	0.68
Age (per year)	0.24	0.06	1.3 (1.1–1.4)	<0.001
Weight (per kg)	−0.22	0.08	0.8 (0.7–0.9)	0.0016
LUT urolithiasis	1.88	0.45	6.5 (2.8–16.7)	<0.001
Breed				0.33
Mixed (referent)	--	--	--	--
Lhasa Apso	1.57	0.89	4.8 (0.9–30.1)	0.079
Bichon Frise	0.33	0.77	1.4 (0.3–6.8)	0.66
Pomeranian	−0.21	0.92	0.8 (0.1–5.1)	0.82
Shih Tzu	−0.29	0.74	0.7 (0.2–3.5)	0.70
Miniature Schnauzer	−0.34	0.73	0.7 (0.2–3.3)	0.64
Standard Schnauzer	−15.9	4343	0.00 (0–25.1)	1.00
Standard Poodle	−14.2	1737	0.00 (0–27.1)	0.99
Doberman Pinscher	−12.2	2121	0.00 (0–524.3)	1.00

LUT, lower urinary tract; --, not applicable for the referent.

## Data Availability

The data presented in this study are available within the article.

## Abbreviations

CaOx	calcium oxalate
LUT	lower urinary tract
UUT	upper urinary tract
